# A probabilistic approach for pediatric epilepsy diagnosis using brain functional connectivity networks

**DOI:** 10.1186/1471-2105-16-S7-S9

**Published:** 2015-04-23

**Authors:** Saman Sargolzaei, Mercedes Cabrerizo, Arman Sargolzaei, Shirin Noei, Anas Salah Eddin, Hoda Rajaei, Alberto Pinzon-Ardila, Sergio M Gonzalez-Arias, Prasanna Jayakar, Malek Adjouadi

**Affiliations:** 1 Department of Electrical and Computer Engineering, Florida International University, Miami, FL 33174, USA; 2 Department of Civil and Environmental Engineering, Florida International University, Miami, USA; 3 College of Innovation and Technology, Florida Polytechnic University, Lakeland, USA; 4 Baptist Health Neuroscience Center, Baptist Hospital, Miami, USA; 5 Brain Institute, Miami Children's Hospital, Miami, USA

## Abstract

**Background:**

The lives of half a million children in the United States are severely affected due to the alterations in their functional and mental abilities which epilepsy causes. This study aims to introduce a novel decision support system for the diagnosis of pediatric epilepsy based on scalp EEG data in a clinical environment.

**Methods:**

A new time varying approach for constructing functional connectivity networks (FCNs) of 18 subjects (7 subjects from pediatric control (PC) group and 11 subjects from pediatric epilepsy (PE) group) is implemented by moving a window with overlap to split the EEG signals into a total of 445 multi-channel EEG segments (91 for PC and 354 for PE) and finding the hypothetical functional connectivity strengths among EEG channels. FCNs are then mapped into the form of undirected graphs and subjected to extraction of graph theory based features. An unsupervised labeling technique based on Gaussian mixtures model (GMM) is then used to delineate the pediatric epilepsy group from the control group.

**Results:**

The study results show the existence of a statistically significant difference (*p *< 0.0001) between the mean FCNs of PC and PE groups. The system was able to diagnose pediatric epilepsy subjects with the accuracy of 88.8% with 81.8% sensitivity and 100% specificity purely based on exploration of associations among brain cortical regions and without a priori knowledge of diagnosis.

**Conclusions:**

The current study created the potential of diagnosing epilepsy without need for long EEG recording session and time-consuming visual inspection as conventionally employed.

## Background

Epilepsy is a neurological disorder characterized by recurrent seizures with unspecified causes. The Center for Disease Control and Prevention (CDC) estimates that more than 2.3 million adults and half a million children in the United States are affected by Epilepsy [1]. This number is projected to dramatically increase every year by about 0.15 million newly diagnosed epilepsy cases [2]. Although the impact of seizures varies from person to person, physical and mental functions of the affected person could be severely altered. A systematic approach for epilepsy diagnosis could improve the planning for a treatment process and thus relieve the burden already imposed on the overall healthcare system. Scalp Electroencephalography (EEG) recording at resting state condition has been widely perceived as an effective preliminary tool for non-invasive study of the brain in individuals with epilepsy. Analysis of Scalp EEG during resting state condition, without performing a cognitive task and with the absence of external stimuli, has gained significant prominence for assessing brain function and related disorders. Applications include tasks that require assessing responses of the brain under the influence of different drug therapies [3], and tasks that rely on determining the 3D source localization of epileptic seizures which exploits techniques in the time/frequency domains for analysis of individual EEG electrode recordings [4,5]. Assessment of brain functional connectivity network in patients suffering with various neurological disorders through modalities such as EEG recording, Magnetoencephalography (MEG) and functional Magnetic Resonance Imaging (fMRI) has elicited new findings in ways of underlying distinctions that delineate epileptic from control populations [6-14]. The high temporal resolution of EEG renders it as an indispensable tool in the primary diagnosis of epilepsy and in visualization of characteristic temporal events like interictal spikes which are closely associated with epileptic foci [7,15]. Additionally, EEG has been utilized to distinguish focal and generalized neurophysiologic correlates of epilepsy [16]. Diagnosis of epilepsy by the means of scalp EEG visual inspection often involves long term recording and investigation by the EEG expert to search for abnormal activities.

However, visual inspection and interpretation of continuous temporal EEG recordings is tedious, time consuming and prone to human error. Furthermore, epilepsy diagnosis based on visual inspection of EEG has been shown to be very subjective to the expert opinion [17]. This has led to the general cohort of adopting various computer aided techniques with the help of machine learning for medical applications [18-20]. Artificial Neural Network (ANN) based classifiers have received the most attention towards epilepsy diagnosis using scalp EEG recordings [21-23] where the accuracy rate of 0.92 [21] and 0.8 [22,24] are reported which involved the existence of training set and a priori knowledge. The general routine of ANN based techniques is to process each isolated EEG signal with the aim of extracting a set of discriminating features as input to train an ANN in the design of an optimal classifier and predictor of the diagnosis. Considering the fact that Epilepsy is a complex disease with multifactorial causes, makes the diagnostic process much more complicated than simply relying on solely model driven knowledge. Furthermore, the human brain includes a complex web of neuronal interconnectivity and discrete anatomical regions that function together to generate brain activity [11,25]. This underlying functional brain infrastructure suggests that a methodology for enhanced epilepsy diagnosis needs to consider the whole brain network as described by its patterns of functional connectivity networks (FCNs). Thus, FCNs seek to define the patterns of cross-correlation between discrete functionally characterized brain regions to give statistical importance to anatomical connectivity (upon the existence of physical connection) and subsequently determining inter-regional neurophysiological inferences. FCNs could be grouped into two broad categories: Directed and Undirected. Undirected FCNs finds the dynamic associations among functional brain regions without considering the hypothetical causalities among them. Whereas Directed FCNs, sometimes referred to as effective connectivity [26], assesses the influence of one cerebral region upon another and therefore gives direction to the calculated associations. Current trends in adopting FCNs for understanding the complex brain are placed toward developing data driven methodologies for constructing FCNs which benefits from a robust parcellation of functional data of the brain and an objective formulation of the hypothesized association among functional parcels [6,26]. The crucial role of time delay in the dynamics of large scale networks [27] such as brain networks is well motivated, due to the large scale property of brain connectivity networks including discrete sub-networks [28], but yet not fully understood and incorporated in constructing the brain networks and decision making processes [29]. Time delay is coupled with the fact that on large scale networks such as brain networks, the recorded signal at each electrode is in fact showing the summation of the variance of the brain area closer to the electrode and a lagged version of variances from other brain areas.

**Figure 1:**
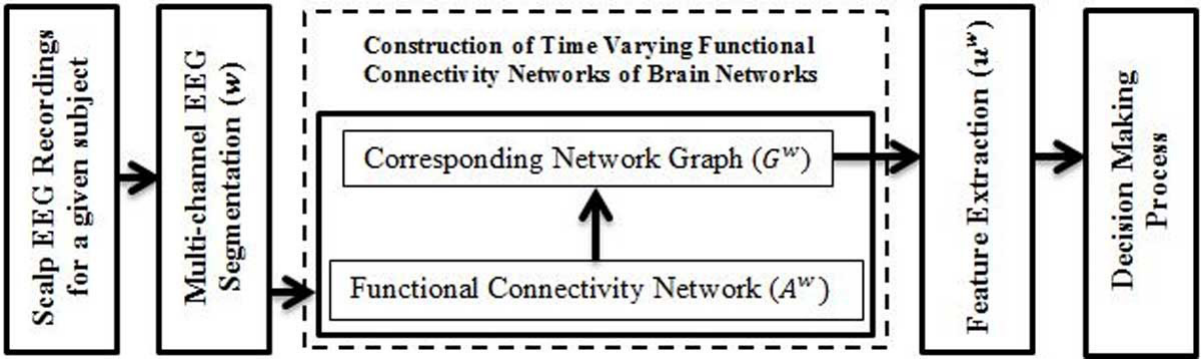
**Flowchart of the proposed decision support system**. Flowchart of the proposed decision support system for computer aided diagnosis of pediatric epilepsy based on machine learning techniques applied on constructed FCNs of the brain. Algorithm starts with segmentation of multichannel EEG recordings by applying a moving window (*w*) with overlap. Functional connectivity networks are constructed and mapped into a corresponding graph for each window. Extraction of graph theoretical based features (*u_w_*) is then followed by a decision making process which uses a probabilistic approach to determine whether a patient is epileptic or not.

In this study, a new algorithm based on time-varying associations among channels of scalp EEG using a moving window is examined for its ability to identify multichannel EEG segments with epilepsy driven characteristics without a priori knowledge assumed about the diagnosis. Undirected FCNs estimate the association between the channels of EEG using a geometrical distance between a pair of channels. Undirected FCNs can be represented in the form of undirected graphs. Each electrode is considered as a node and the functional associations among them are the edges of the corresponding graph. Different applications of graph theory and small world networks [30], with causality analysis combined with network analysis [31] and time-frequency coupling detection among isolated scalp EEG recordings [32,33] are a few of the widely used model-based solutions for studying FCNs using scalp EEG recordings. This study also assesses the merit of graph theory based features, extracted from the graphs corresponding to the FCNs, in identifying EEG segments recorded from patients with epilepsy from segments recorded from the pediatric control group. The main contribution of the study is in introducing a novel approach based on purely machine learning techniques to facilitate the screening process of potential epileptic patients. The emphasis of developing the new decision support system for pediatric epilepsy diagnosis was placed on the accurate diagnosis without a priori knowledge. The probabilistic decision on the subject diagnosis makes the system more applicable in clinical environments. An overview of the proposed decision support system for computer aided diagnosis of pediatric epilepsy is as shown in Figure 1.

## Methods

### Subjects and data

Scalp EEG recordings from 18 pediatric patients (7 pediatric control (PC) group and 11 pediatric epilepsy (PE) patients) were included in this study. The study was approved by the Institutional Review Board (Protocol number: IRB-052708-03) and consent forms were provided to the patients or legal representatives. The scalp EEG data were collected from routine EEG recordings without the imposition of long recording sessions. Recordings were performed using XLTEK Neuroworks Ver. 3.0.5 equipment following 10-20 electrode placement system (referential montage) and sampled, indistinctly for PC and PE subjects, at varying frequencies of 200 Hz, 500 Hz and 512 Hz. Segments free of artifacts, 4.2 seconds to 90 seconds long, from all EEG recordings were initially selected. Care was given to the initial selection of EEG segments such that they contained no seizure activity to prevent any bias. The inclusion of interictal spikes, as a representation of cortical irritability, is based on the fact that they could be observed in normal control subjects without being considered as epilepsy segments [21]. Due to non-stationary characteristics of long recordings of EEG signal [21,34], and for an unbiased evaluation of the study results, initial intervals were broken into segments with equal length by applying a moving window with the length of 9 seconds and 4.5 seconds overlap. The choice of 9-second window length was based on the EEG segmentation strategy using the energy function calculated using the Teager's algorithm [34,35]. An overview of demographic characteristics of study subjects is given in Table 1.

**Table 1:** Demographic characteristics of study subjects

			Age	Female/Male	Number of Segments
**PC (n = 7)**			12.86 ± 3.39*	3/4	13 ± 6.98

**PE (n = 11)**			9.09 ± 4.81	5/6	32.18 ± 22.41

p**			ns	ns	< 0.05

***Subject ID***	***Age***	***Gender***	***Diagnosis***	***Sampling Rate (Hz)***	***Number of Segments***

**PC01**	12	M	-	200	11

**PC02**	15	F	-	512	20

**PC03**	12	M	-	200	14

**PC04**	15	F	-	512	18

**PC05**	10	M	-	512	3

**PC06**	18	F	-	512	20

**PC07**	8	M	-	512	5

**PE01**	10	F	Left temporal dysplasia	200	14

**PE02**	7	F	Left frontal region	512	67

**PE03**	4	F	Right fronto-centro-temporal	512	39

**PE04**	14	M	Generalized	512	18

**PE05**	8	M	Right parietal	200	30

**PE06**	7	M	Left frontal pole, posterior frontal lobe	512	77

**PE07**	15	F	Left and right frontal	500	40

**PE08**	4	M	Right fronto-centro-temporal	512	25

**PE09**	2	F	Left temporal (posterior)	512	25

**PE10**	14	M	Generalized	512	6

**PE11**	15	M	Generalized	512	13

*Data Presented as mean ± S.D. where applicable

**Student t test (with statistical significance threshold of 0.05) was used to test for age and number of segments.

Fisher's exact test was used to test for gender.

PC = Pediatric Control; PE = Pediatric Epilepsy;

M: Male; F: Female.

ns: not significant.

### Functional connectivity networks construction

The aim of functional connectivity of brain networks construction is to determine the existing pattern of functional association among hypothetically isolated channels and the causality relationship between anatomical brain regions. Emphasis is placed on the connection topology in the functional layer rather than on discovery of the existence of structural medium among cortical regions. Identifying the frequency based pattern of information propagation between brain regions to study the epileptic discharges has been recently investigated [21]. This study focuses on exploring the relationship among brain regions based on the time-domain electrical activities recorded using multi-channel scalp EEG. Time-domain characteristics of seizures and epileptic form discharges [34] enforce the computer aided diagnosis systems to look over short segments of EEG. Studying the dynamics of the neural networks within the cerebral cortex at higher time resolution increases the accuracy of interictal spike detection and 3-D source localization of seizures [21,36]. The proposed solution explores FCNs using multi-channel EEG segments (9 seconds long [34]), which through a geometrical approach, the time-varying patterns of FCNs are assessed. FCNs are calculated in the form of a two dimensional array called adjacency matrix, *A^w^_und_*, as defined in equation (1), where *w *defines a segment of multi-channel EEG data and with subscript und denoting the undirected from. Elements of adjacency matrix are the pairwise strength of connectivity among scalp EEG recording channels for the segment *w*.

(1) $${A^{w}}_{wnd}={\left[{\theta_{ij}}^{w}\right]}_{m\times m}\;\;i,j=1,...,m$$

(2) $${\theta_{ij}}^{w}=\pi -cos^{-1}\left(\frac{\sum_{n=1}^{N}x_{i}\left[n\right]x_{j}\left[n\right]}{\sqrt{\sum_{n=1}^{N}{x_{i}}^{2}\left[n\right]}\sqrt{\sum_{n=1}^{N}{x_{j}}^{2}\left[n\right]}}\right)\;\;i,j=1,...,m$$

Connectivity strength, which establishes a symmetric adjacency matrix. Following the symmetric property of undirected FCN, a geometric distance as shown in equation (2) is used to calculate the pairwise connectivity strength. The proposed geometric distance is a modification of the cosine similarity metric.

Each *θ_ij_^w ^*value represents the pairwise connectivity strength between electrodes *i *and *j *for the corresponding window *w *with their electrical activity recordings denoted as *x_i_*[*n*] and *x_j_*[*n*], respectively. The value of *n *represents the discrete time sample number which ranges from 1 to *N *where *N *is the length of window *w*, and *m *is the number of electrodes considered for constructing the FCNs. The angular value of *θ_ij_^w ^*of 0 radian specifies the highest connectivity strength which is most likely the case when calculating the distance among an electrode and itself, and a value of $\frac{\pi }{2}$ radian shows that the respective pair of electrode recordings is orthogonal and corresponds to the maximum distance (i.e., lowest connectivity strength).

### Graph mapping of brain networks and feature extraction

Graph theory is a well-established and rich source of benchmarks for studying functional connection as well as anatomical connections in the brain [37]. Directed FCNs (*A^w^_dir_*) and undirected FCNs (*A^w^_und_*) in terms of connectivity strength among pair of scalp EEG channels recordings could be mathematically represented and studied in the forms of directed and undirected network graphs [25]. Graph *G^w^*, for a given segment *w*, is comprised of a set of vertices, *V^w^*, and a set of edges, *E^w^*. In the context of FCNs, vertices are the scalp EEG electrodes and their location in the graph can be determined from the coordinate of the electrode position in the 10-20 electrode placement system space. Similarly, the graph edges are the hypothetical functional connection among the vertices and the connection strengths calculated as in equations (1) and (2) define the weights of these edges. In mapping these FCNs, it is hypothesized that the small world network model of brain function could be altered by epilepsy [10,38,39]. These hypothesized alterations could be used to consequently discriminate a network graph corresponding to a patient with epilepsy from that of a control group. Network graph *G^w ^*based statistics (Table 2) [40] are calculated for each segment *w *to form the feature vector *u^w ^*as shown in equation (3):

**Table 2:** Graph theoretical features of functional connectivity networks

Feature	Feature description	Feature calculation
ldg	Link density of the graph	(2 × *ne*)/(*nn *× (*nn *- 1))

acc	Average of closeness centrality	(1/*nn*) ∑*_nn_*(*sum of reciprocal distances from a node to all other nodes*)

gcc	Graph clustering coefficient	(3 × *number of triangles*)/(*number of connected triples of nodes*)

rcc	Rich club coefficient	(*ne*_*k*)/(*nn*_*k *× (*nn_k _*- 1))

smg	S-metric of graph	*Sum of the nodal degree products for every edge*.

acg	Algebraic connectivity of graph	*Second smallest eigenvalue of the Laplacian of adjacency matrix*.

eng	Energy of network graph	*Sum of absolute values of the real components of eigenvalues of adjacency matrix*.

*ne*: Number of graph edges; *nn*: number of graph nodes;

*nn_k*: Number of nodes with degree larger than*k*; *ne_k*: number of edges among nodes with degree larger than *k*.

(3) $$u^{w}={\left[\begin{array}{ccccccc} ldg & acc & gcc & rcc & smg & acg & eng \end{array}\right]}^{T}$$

### Decision making process

**Figure 2:**
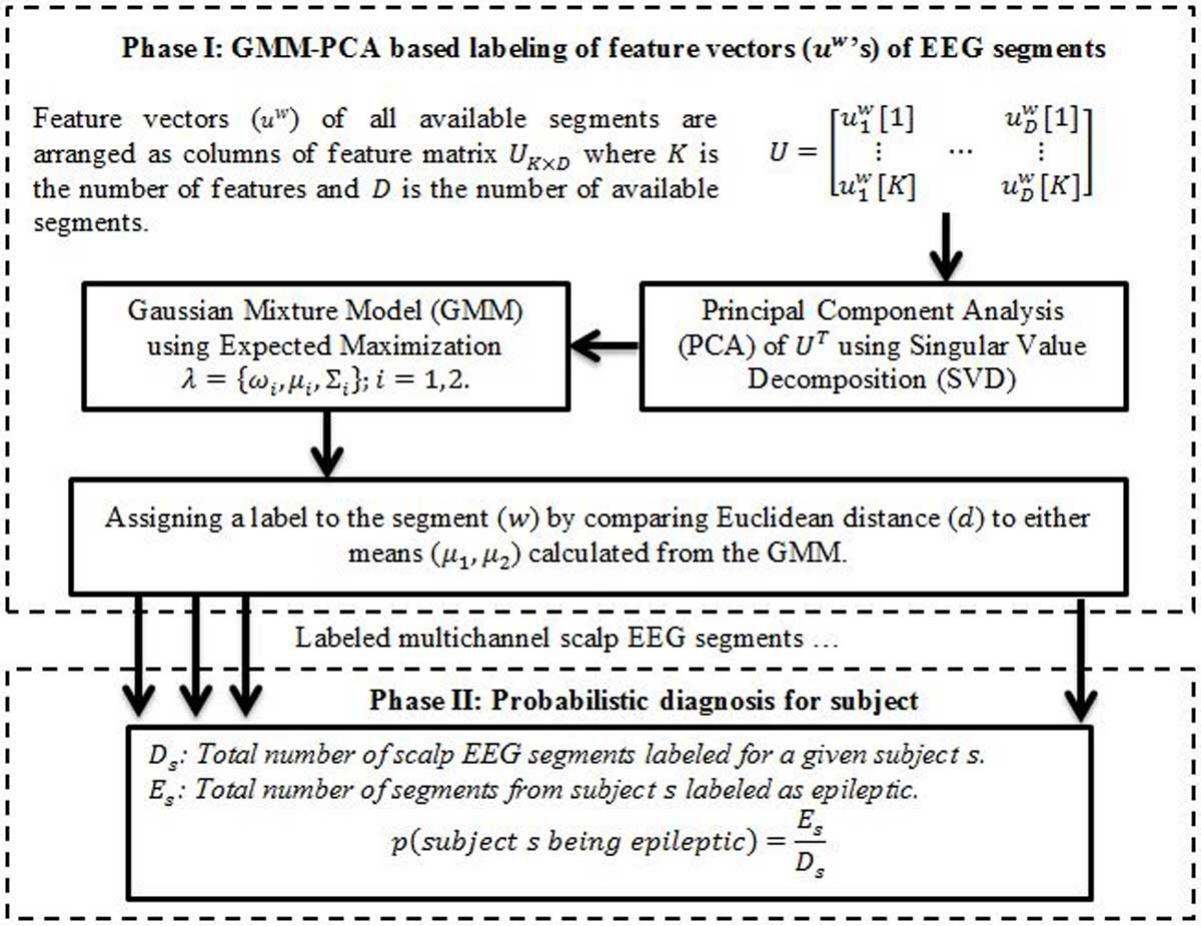
**Flowchart of the two phase decision making process**. Flowchart of the two-phase decision making process. Phase I assigns labels to multichannel scalp EEG segments based on the Gaussian Mixture Modelling of graph theoretical based features; Phase II assigns labels (epileptic or non-epileptic) to the subjects by assigning a probability in the likelihood of belonging in one of the two groups.

As the multichannel scalp EEG is broken into segments (*w*) and analyzed to generate the feature vectors (*u^w^*), when using a training set with annotated EEG segments (epileptic or non-epileptic), a classifier could be trained to make a decision on a newly recorded EEG segment in the testing phase. However in the absence of training data and/or a priori diagnosis, machine learning techniques could be instead incorporated to make an optimized decision on the nature of segment whether it belongs to the PE or PC group. One of the merits of the current study is in the ability to identify epileptic EEG's from non-epileptic EEG when no prior knowledge is at hand. The proposed system as designed consists of two phases of decision making. Figure 2 shows the steps in making a decision.

#### Gaussian Mixture Model (GMM) for EEG Segments Labeling

Input to the decision making block is a feature matrix, *U*_*K*×*D*_, whose columns are the feature vectors corresponding to the segments, where *K *is the number of features and *D *is the total number of segments. This feature matrix is then subjected to the principal component analysis (PCA) using singular value decomposition (SVD) [41]. This step is to map the covariance of the data into a new coordinate whose bases express the most variance. The output of PCA using the first principal component, *x*_1 × *D*_, is considered as the probability distribution of graph theoretical features (*u^w^*) across all segments. *x*_1 × *D *_is then modeled parametrically using a Gaussian Mixture Model (GMM) using the two mixtures *λ *= {*ω_i_*, *μ_i_*, Σ*_i_*}; *i *= 1,2 as shown in equation (4).

(4) $$p\left(x|\lambda \right)=\sum_{i=1}^{2}\omega_{i}g\left(x|\mu_{i},{\text{Σ}}_{i}\right)$$

Where *ω_i _*elements are the mixture weights, and *g*(*x*|*μ_i_*, Σ*_i_*) represents the Gaussian densities calculated from *D*-variate Gaussian distribution with parameters *μ_i _*and Σ*_i _*(*i *= 1,2) as their respective means and covariance matrices [41-43]. Parameters of the model were estimated through maximum likelihood (ML) estimation method and EEG segments are then labelled based on their closeness to the either estimated means.

#### Probabilistic approach in the decision making process

The decision support system developed in the current study serves as an auxiliary tool in clinical settings. Therefore providing the neurologists with a probability of the risk factor for a given subject is more preferable rather than logical decisions (epileptic vs. non-epileptic). The number of segments labeled as epileptic for a specific subject is used in phase II of the decision making process to assign a probability as shown in equation (5) on the evidence of these segments' labeling.

(5) $$p\left(subject\;is\;epileptic\right)=\frac{E_{s}}{D_{s}}$$

Where *E_s _*is the number of segments from the given subject being labeled as epileptic out of total number of segments *D_s _*for the corresponding subject.

## Results and discussion

**Figure 3:**
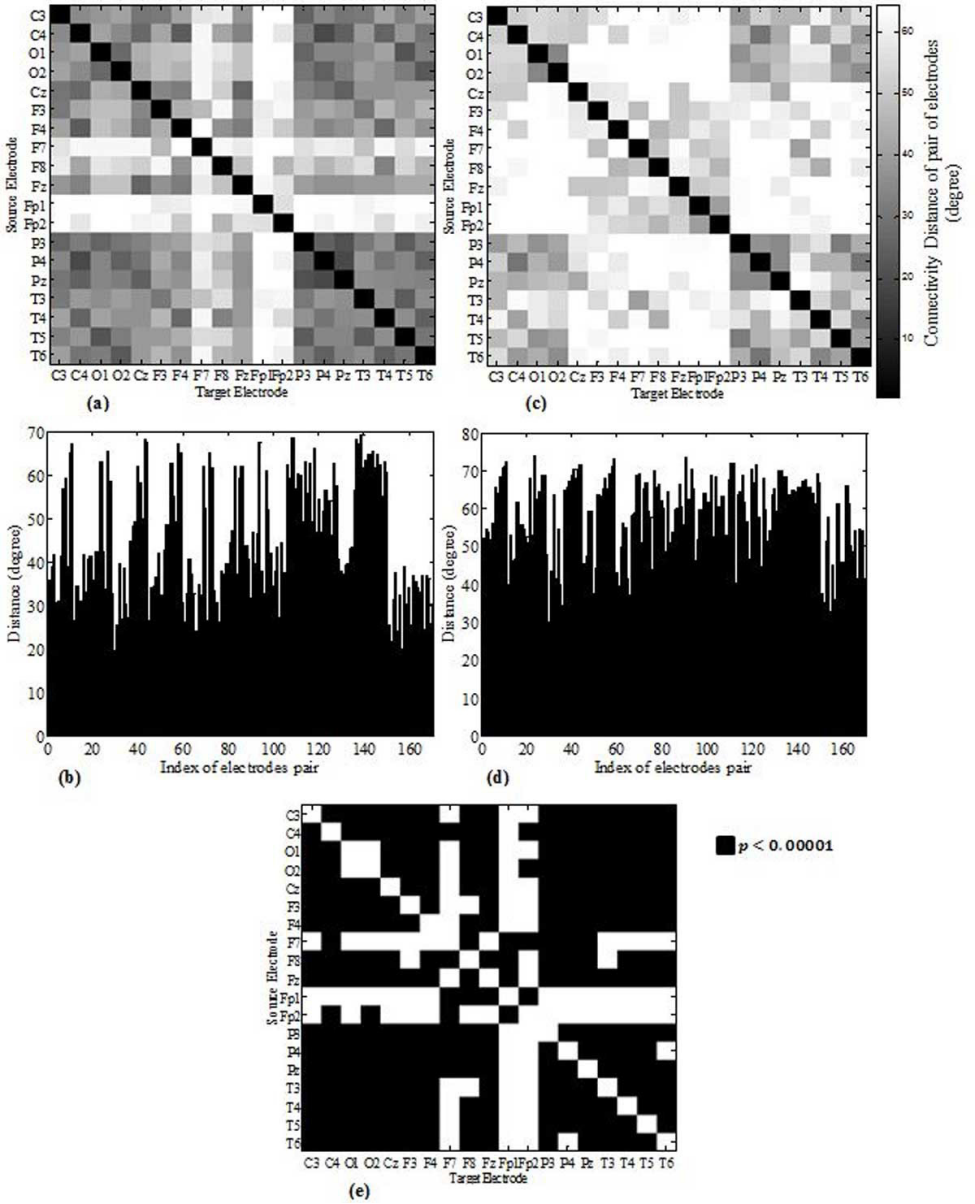
**Visualization of constructed functional connectivity networks**. Visualization of constructed undirected functional connectivity networks (FCNs) and the corresponding plot of the connectivity distances for the average map across (a), (b) pediatric control group (c), (d) pediatric epilepsy. Index of electrodes pair represent the pair of electrodes, e.g. index 0 corresponds to the connectivity distance (angle) among pair c3-c4 and the index 1 corresponds to the connectivity distance (angle) among pair c3-o1 and etc. (e) shows the results of student t test for the null hypothesis that assumes no statistically significant differences for the index pair of electrodes across the pediatric epilepsy and pediatric control groups. Rejection of the null hypothesis is highlighted with the black boxes when *p *< 0.00001, bonferroni adjusted for multiple comparison.

Figure 3 shows the constructed undirected FCNs averaged for both pediatric epilepsy (PE) and pediatric control (PC) groups with their corresponding bar plots of connectivity distances (degrees) and under different degrees of strengths. The symmetric property of the adjacency matrices is due to the nature of the constructed FCNs which do not take into consideration the possible causality among brain cortical region. The change in the pattern of functional associations is observed by increasing the distances among some brain regions such as cz-o1 and cz-o2 pairs. A two-sided two sample student t-test was conducted to assess whether there is a significant difference between the undirected mean FCNs of the PC group and the PE group. The test results indicated that there is a statistically significant difference (*t*(340) = -9.89, *p *< 0.0001) between the mean FCNs of the PC group (*μ_PC _*= 44.56°, *σ_PC _*= 13.75° *σ_PC _*= 13.75°) and the PE group (*μ_PC _*= 57.74°, *σ_PE _*= 10.70°). Furthermore, we performed a two samples student t test to inspect the existence of statistical significant difference among each connection between source electrode and target electrode (index of electrode pair in Figure 3(b) and 3(d)) across the pediatric control and pediatric epilepsy group. Results are visualized in Figure 3(e). Existence of statistically significant (*p *< 0.00001 Bonferroni adjusted for multiple comparisons) difference in the connection strength (degree) of a specific source-target pair of electrodes is shown as a black box unit, insignificant difference in the connection strength is shown as a white box unit. As the large number of black boxes in Figure 3(e) states, the brain acts as a complex network and epilepsy affects more than single connection. However this needs to be more investigated on a subject basis as the type of epilepsy (focal or generalized) could vary from subject to subject.

**Figure 4:**
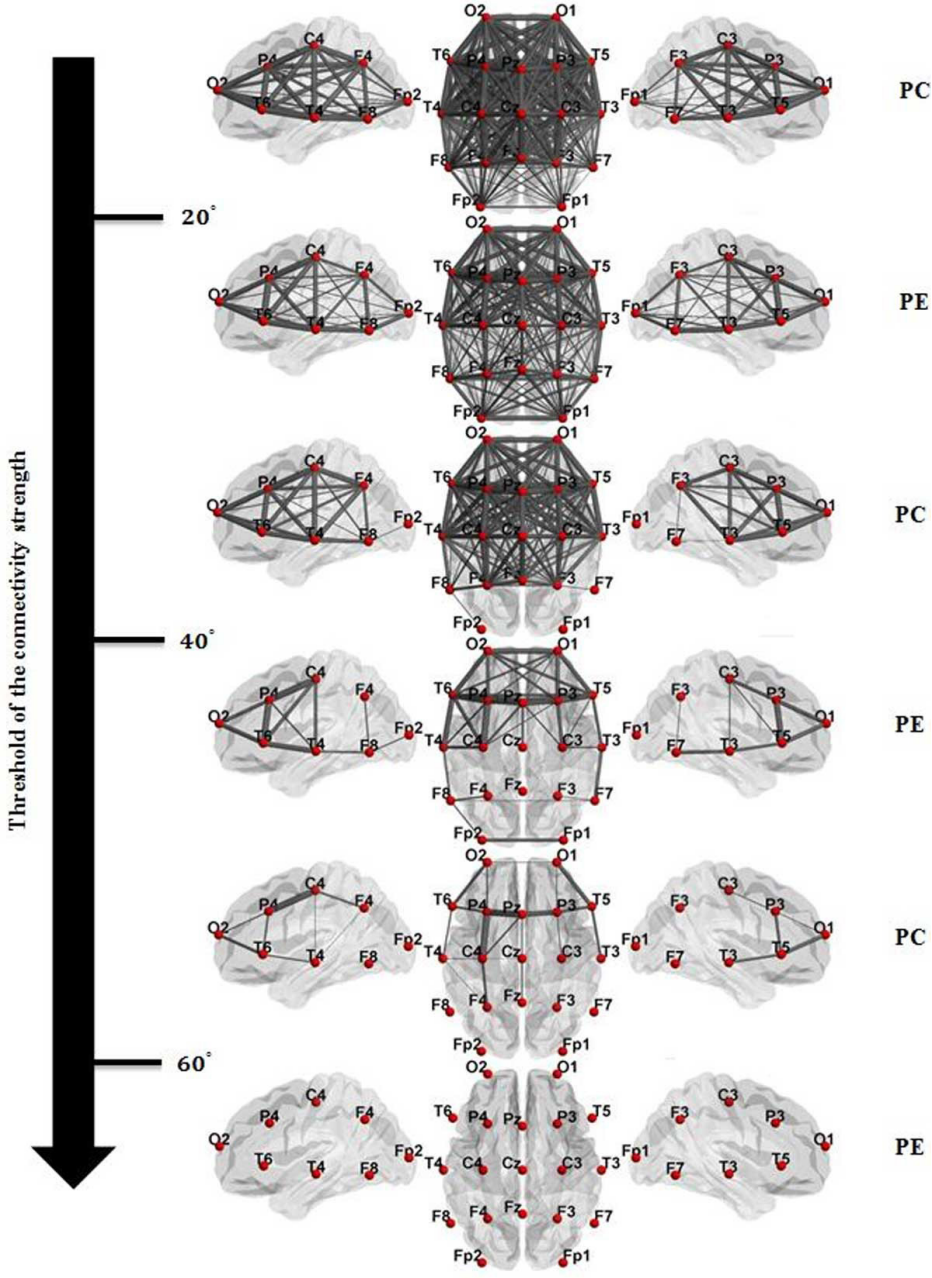
**Graph representations**. Graph representations of average of constructed FCNs over the (a) pediatric control (PC) group and (b) pediatric epilepsy (PE) group. Thickness of links (graph edges) shows the strength of connectivity among electrode pairs.

Adjacency matrices shown in Figure 3 are mapped into the graph representations shown in Figure 4. Analysis of the single connections of the graphs is beyond the scope of this study; however the reduction in the pattern of inter-connections among isolated brain regions is visually observable by comparing the two graphs; Table 3 provides the statistics of the features extracted for both graphs and tests for the differences among PC and PE groups. We also performed a connection density assessment on left and right hemispheres and the interconnection among the hemispheres across subjects. Density of connections on each hemisphere were calculated by counting the number of edges with the weight less than or equal to 45° (mid-point in connectivity strength). The results summarized in Table 4 show alterations in the wiring pattern of the brain functional network caused by epilepsy. This alteration is in the form of reduced density of connectivity in both left and right hemisphere as well as inter-hemispheric connectivity. Note also how the connectivity maps for both the PE and PC groups as the threshold of the connectivity strength is changed.

**Table 3:** Statistical analysis of features across PC and PE

Feature	PC	PE	p
***ldg***	44.56 ± 7.77**	57.74 ± 8.44	< 0.000

***acc***	0.0014 ± 0.0003	0.0010 ± 0.0003	< 0.000

***gcc***	1.18 ± 0..006	1.18 ± 0.005	*ns*

***rcc***	44.56 ± 7.77	57.74 ± 8.44	< 0.000

***0smg***	2.38 ± 0.81	3.98 ± 1.04	< 0.000

***acg***	659.76 ± 135.45	913.24 ± 169.46	< 0.000

***eng***	1.68 ± 0.26	2.11 ± 0.28	< 0.000

*Two-way Student t test is used for test the difference between PC and PE groups. Statistical significant level of 0.01 is considered for p-value.

**mean ± standard deviation.

**Table 4:** Connectivity strength for left hemisphere, right hemisphere and inter-hemispheres

	Hemisphere Connection				Hemisphere Connection		
	**Right**	**Left**	**Inter-hemisphere**		**Right**	**Left**	**Inter-hemisphere**

**PC01**	10.82 ± 2.6	9.09 ± 4.23	18 ± 11.4	**PE01**	10.64 ± 4.41	3.42 ± 2.79	10 ± 7.1

**PC02**	19.3 ± 4.12	19.55 ± 4.16	34.1 ± 12.45	**PE02**	9 ± 3.39	7.75 ± 2.3	9.75 ± 5.98

**PC03**	20 ± 1.62	15.71 ± 1.14	33.42 ± 5.62	**PE03**	11.87 ± 4.93	16.28 ± 6.1	26.13 ± 15.4

**PC04**	16 ± 5.72	15.5 ± 4.74	27 ± 18.82	**PE04**	15.28 ± 4.84	13.11 ± 7.5	22.16 ± 17.5

**PC05**	24.67 ± 1.5	15 ± 1.73	33.33 ± 16	**PE05**	9.6 ± 6.5	11.5 ± 5.3	14 ± 16.2

**PC06**	18.7 ± 2.56	15.2 ± 3.67	35.75 ± 7.57	**PE06**	9.7 ± 1.38	8.5 ± 1.7	19.25 ± 4.78

**PC07**	13.8 ± 3.96	12.8 ± 3.96	27 ± 11.62	**PE07**	9.85 ± 1.72	11.97 ± 4.4	17.15 ± 9.1

**Pooled statistics**	**17.4 ± 3.45**	**15.42 ± 3.64**	**30.59 ± 11.5**	**PE08**	6.28 ± 2.82	7.7 ± 5.73	9.16 ± 11.43

				**PE09**	14.36 ± 3.36	5.1 ± 1.1	4.64 ± 1.87

				**PE10**	5.6 ± 3	7 ± 4.7	11.17 ± 7.3

				**PE11**	14.15 ± 1.72	15.38 ± 5	34 ± 7.85

				**Pooled statistics**	**10.32 ± 3.19**	**9.82 ± 3.64**	**15.97 ± 8.77**

For the evaluation purposes, no information was provided for multichannel EEG segments in terms of diagnosis (epileptic vs. non-epileptic) of the subject to whom the segments belong to. Results of labelling procedure using Gaussian mixture model (GMM) and the probability of the corresponding subject being epileptic are given in Table 5. The suggested decision making system based on GMM has shown the ability of detecting epileptic segments with an accuracy of 81.3% with 77.4% sensitivity and 96.7% specificity solely based on discovery of the hypothetical associations among cortical regions. The probability approach shows the power of the proposed algorithm in decision making based on the segment labeling and time-varying FCNs as examplified in subjects PE04 and PC04. Both subjects have segments labeled as the contrary group which the subject indeed belongs to; however the probability approach based on the time-varying FCNs identifies them correctly when the decision is made. The mis-identification of labels in subjects PE03 and PE11 could not be corrected by the suggested probability approach.

**Table 5:** Clustering results with no prior knowledge provided on diagnosis

	Condition		
	**Healthy**	**Epileptic**	

Clustered as Epilepsy	0	9	Positive predictive value (%) 100

Clustered as Healthy	7	2	Negative predictive value (%) 77.8

	Sensitivity (%) 81.8	Specificity (%) 100	Accuracy (%) 88.9

**Subject ID **	***E_s_***	***D_s_***	**Probability (%)**

**PC01**	0	11	0

**PC02**	0	20	0

**PC03**	0	14	0

**PC04**	2	18	11

**PC05**	0	3	0

**PC06**	0	20	0

**PC07**	1	5	20

**PE01**	12	14	86

**PE02**	67	67	100

**PE03**	10	39	25

**PE04**	12	18	66

**PE05**	21	30	70

**PE06**	66	77	86

**PE07**	30	40	75

**PE08**	23	25	92

**PE09**	25	25	100

**PE10**	5	6	83

**PE11**	3	13	23

This mis-identification could be due to different factors such as the window size, number of segments required to accurately diagnose or the type of epilepsy which needs more investigation.

The assumption of the existence of no priori knowledge on the diagnosis in the clinical environment could be relieved by assuming the existence of symptoms and the minimal knowledge of a training set of multi-channel EEG segments which could be considered as a tuning approach in the decision making process. A training set was composed including twenty randomly chosen multi-channel EEG segments from the total set of EEG segments and Support Vector Machine (SVM) with a linear kernel were trained to classify the segments. The testing set was then given to the system after self tuning and the results showed 100% accuracy in classification accuracy of the multi-channel EEG segments.

## Conclusions

A novel decision support system for computer aided diagnosis of pediatric epilepsy using machine learning techniques was presented. The approach taken in the system was based on constructing functional connectivity networks (FCNs) of the brain and analyzing graph theoretical based features to identify the wiring pattern differences among pediatric control (PC) and pediatric epilepsy (PE) groups. The system is designed to provide clinicians with initial screening results about the likelihood of a given subject to be epileptic or not. The time-varying FCNs implementation increases the resolution by segmenting the multichannel EEG. This created the potential of diagnosing epilepsy without need for long EEG recording session and time-consuming visual inspection as conventionally employed. The main contribution of the study is the reliance of the algorithm on machine learning techniques to facilitate the screening process of potential epileptic patients without need of a priori knowledge and without need for a training phase. The suggested window length in constructing FCNs, the number of principal components (dimension of GMM) to be used, and the inspection of possible causal relationships among cortical brain regions are areas that need further investigation on the basis of a larger dataset.

## Competing interests

The authors declare that they have no competing interests.

## Authors' contributions

Developed and implemented the study algorithm: SS, AS, MC, SN, AS, MA. Performed the experiments and designed the study protocols: SS, AS, SN, HR, AP, SM, PJ, MA. Processed the data, prepared the tests, analyzed and interpreted the study results: SS, AS, SN, MA. Performed statistical analyses: SS, AS, MC, HR, PJ, MA. Drafting of the manuscript: SS, AS, MC, MA. All authors read and approved the final manuscript.

## Declarations

The publication costs for this article were partially funded by the FIU Open Access Publishing Initiative.

This article has been published as part of *BMC Bioinformatics *Volume 16 Supplement 7, 2015: Selected articles from The 11th Annual Biotechnology and Bioinformatics Symposium (BIOT-2014): Bioinformatics. The full contents of the supplement are available online at http://www.biomedcentral.com/bmcbioinformatics/supplements/16/S7.
